# Chitosan Nanoparticles as a Mucoadhesive Drug Delivery System for Ocular Administration

**DOI:** 10.3390/md15120370

**Published:** 2017-12-01

**Authors:** Mariana M. Silva, Raquel Calado, Joana Marto, Ana Bettencourt, António J. Almeida, Lídia M. D. Gonçalves

**Affiliations:** 1 Research Institute for Medicines (iMed.ULisboa), Faculty of Pharmacy, Universidade de Lisboa, 1649-003 Lisbon, Portugal; silva.mariana@campus.ul.pt (M.M.S.); raquel.calado@gmail.com (R.C.); jmmarto@ff.ulisboa.pt (J.M.); asimao@ff.ulisboa.pt (A.B.); aalmeida@ff.ulisboa.pt (A.J.A.); 2 Laboratório Edol, Produtos Farmacêuticos S.A., 2795-225 Linda-a-Velha, Portugal

**Keywords:** mucoadhesion, nanoparticles, ocular, ceftazidime, viscosity

## Abstract

Pharmaceutical approaches based on nanotechnologies and the development of eye drops composed of the mucoadhesive polymers chitosan and hyaluronic acid are emerging strategies for the efficient treatment of ocular diseases. These innovative nanoparticulate systems aim to increase drugs’ bioavailability at the ocular surface. For the successful development of these systems, the evaluation of mucoahesiveness (the interaction between the ocular delivery system and mucins present on the eye) is of utmost importance. In this context, the aim of the present work was to investigate the mucoadhesivity of a novel nanoparticle eye drop formulation containing an antibiotic (ceftazidime) intended to treat eye infections. Eye drop formulations comprised a polymer (hydroxypropyl) methyl cellulose (HPMC) 0.75% (*w*/*v*) in an isotonic solution incorporating chitosan/sodium tripolyphosphate (TPP)-hyaluronic acid-based nanoparticles containing ceftazidime. The viscosity of the nanoparticles, and the gels incorporating the nanoparticles were characterized in contact with mucin at different mass ratios, allowing the calculation of the rheological synergism parameter (∆η). Results showed that at different nanoparticle eye formulation:mucin weight ratios, a minimum in viscosity occurred which resulted in a negative rheological synergism. Additionally, the results highlighted the mucoadhesivity of the novel ocular formulation and its ability to interact with the ocular surface, thus increasing the drug residence time in the eye. Moreover, the in vitro release and permeation studies showed a prolonged drug release profile from the chitosan/TPP-hyaluronic acid nanoparticles gel formulation. Furthermore, the gel formulations were not cytotoxic on ARPE-19 and HEK293T cell lines, evaluated by the metabolic and membrane integrity tests. The formulation was stable and the drug active, as shown by microbiological studies. In conclusion, chitosan/TPP-hyaluronic acid nanoparticle eye drop formulations are a promising platform for ocular drug delivery with enhanced mucoadhesive properties.

## 1. Introduction

Ocular infections may threaten vision, and the classically developed drug delivery systems fail to solve the primary problem [1,2]. *Pseudomonas aeruginosa* is among the principal pathogens responsible for corneal ulcers. Ceftazidime (CFT) is currently the most active cephalosporin available against *P. aeruginosa*, and is resistant to various β-lactamases. However, ceftazidime undergoes rapid degradation in aqueous solutions, resulting in the opening of the β-lactam ring. Because of its instability, eye drops containing ceftazidime in aqueous solutions are not commercially available [3]. 

The majority of ophthalmic formulations are administered as eye drops due to their ease of use, safety, and simplicity of formulation. Nevertheless, there are several mechanisms in the eye that are responsible for keeping the ocular surface free from foreign substances and for washing off most of the drug before they are able to penetrate the required tissue (e.g., rapid nasolacrimal drainage, lacrimation, and tear dynamics) [4,5]. As a result, less than 5% of the administered drug penetrates the cornea to reach intraocular tissues. Therefore, conventional eye drops require frequent instillation of highly concentrated solutions to achieve the therapeutic effect, which may induce ocular and systemic side effects and cellular damage in the eye surface [6,7,8,9]. For these reasons, it is critical to work towards the improvement of the ocular penetration, bioavailability, and effectiveness of the drug in the ocular surface. 

In order to lengthen the ocular residence time of the drug and improve bioavailability, several authors have proposed the introduction of new drug delivery systems using mucoadhesive polymers. The use of mucoadhesive polymers in the form of nanoparticles is a potential strategy to prolong the residence time and bioavailability of the encapsulated drug in the ocular surface. Because of their increased viscosity, they can adhere to the eye and reduce the drug drainage rate [10,11]. 

Considering these facts, our group focused on the formulation of polymeric nanoparticles (NP) for ceftazidime delivery, comprising the two mucoadhesive polymers chitosan (CS) and hyaluronic acid (HA), with sodium tripolyphosphate (TPP) as crosslinking agent.

The polysaccharide chitosan is derived from chitin deacetylation, and can be obtained industrially by the hydrolysis of the aminoacetyl groups of chitin from crabs or shrimps in aqueous alkaline solutions [12]. CS has several features which make it favorable for controlled drug delivery to the eye. It is a hydrophilic mucoadhesive polymer which is biocompatible and biodegradable, with good eye tolerability. Moreover, CS exhibits antimicrobial and wound healing properties [13]. Indeed, the positively-charged amino groups of CS interact with the negatively-charged sialic acid residues of mucin, extending the corneal contact time of the drug [5,14]. Hyaluronic acid (HA) is a natural glycosaminoglycan present in the extracellular matrix of connective tissues in vertebrates, such as the vitreous and aqueous humour in the eye [13]. It is widely used in ocular drug delivery systems because of its interesting characteristics like mucoadhesiveness, biocompatibility, and biodegradability, interacting selectively with cell-surface HA receptors such as CD44, which are present in the ocular epithelium and promote wound healing [15,16]. Thus, our hypothesis was that the combination of these polymers would promote mucoadhesion and prolong the drug’s therapeutic action.

Some previous publications refer to the use of CS and HA in NP for ocular drug delivery. Positive results were obtained with the use of CS, showing that the NP interacted preferentially with the ocular epithelium when compared to a control solution during 24 h, supporting their use for controlled drug release [5]. De la Fuente et al. (2010) [15,16] designed NP composed of HA and CS for the efficient encapsulation of different protein drugs. Further studies with HA-CS NP revealed good in vivo tolerability at ocular surface structures and high capacity to interact with and penetrate the ocular epithelium [17]. 

The goal of our study was to prepare and evaluate an eye drop formulation containing CS/TPP/HA NP for the delivery of ceftazidime into the eye. To the best of our knowledge, there is no reference in the literature showing the potential of these nanoparticulate systems for the successful ocular administration of antibiotics. 

## 2. Results

### 2.1. Preparation and Characterization of CS/TPP/HA NP Formulations

A (hydroxypropyl) methyl cellulose (HPMC) hydrogel 0.75% (*w*/*v*) was selected based on its characteristics, as summarized in Table 1. It can be sterilized in an autoclave, which is a simple, economical, and safe method of easy validation; it presented an adequate viscosity (ca. 5–100 mPa s) and a Newtonian behavior; the pH value of 7.22 is proximal to that of the lacrimal fluid and the osmolality value around 300 mOsm/kg indicates an isotonic solution; the zeta potential (ZP) measurements revealed that HPMC has a non-ionic nature [18]. These results grant maximum comfort of the eyes according to the European Pharmacopeia 9.0 (monograph “Eye Preparations”). As such, HPMC seems to be an adequate polymer for use in ocular administration; in fact, it has been largely used in ophthalmic solutions as a viscosity enhancer to improve the drug absorption [19,20]. 

After preparation, the NP were analyzed for determination of particle size, polydispersity, and ZP. Particle size was within the nanometric range, with a narrow particle size distribution that may be inferred from the polydispersity index values ≤0.4. Surface charge was markedly positive, as shown by the ZP values obtained. The ionotropic gelation method allowed the formation of CS/TPP/HA NP loaded with CFT with a particle size of around 350 nm. Overall, the encapsulation of CFT did not induce any significant changes in size or ZP, which remained suitable for ocular delivery (Table 2). 

### 2.2. In Vitro CFT Release Studies

**Figure 1:**
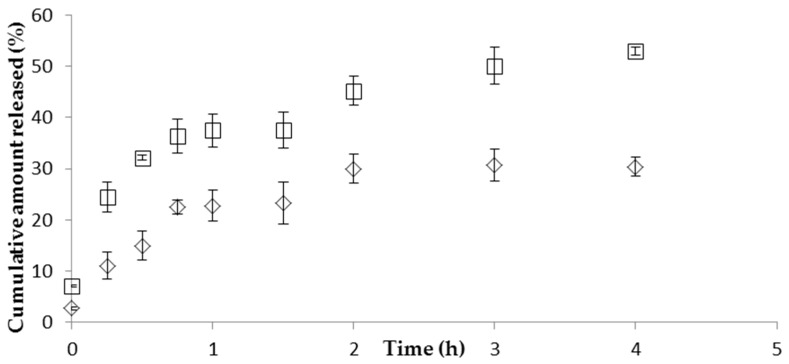
Release profiles of CFT from CS/HA/TPP nanoparticles (in isotonic solution (□) or in hydrogel (◊)) in 10 mM phosphate-buffered saline (PBS) pH 7.4 at 37 °C (mean ± SD, *n* = 3).

The in vitro release profile of CFT revealed a continuous drug release from the NP over 2 h in a pH 7.4 phosphate-buffered saline (PBS) medium. The incorporation of the NP formulation in the hydrogel resulted in a decrease of both the release rate and the total amount of CFT released (Figure 1). The NP released almost 50% of drug in the first 4 h, whereas the gel formulations released only about 30% of drug in the same period. These values remained stable over 24 h (data not shown). The drug release kinetics was characterized by fitting the experimental data with the standard release equations, Equations (3)–(7). The best fitted model for the release data for NPs in isotonic solution or in hydrogel, showing the highest determination coefficient (*r*^2^), was the Korsmeyer–Peppas (Table 3), suggesting that more than one type of mechanism is involved in the release of CFT from the formulations. Moreover, the Korsmeyer–Peppas release model exponent, *n*, was ≤0.5 for both formulations, indicating that Fickian diffusion is the controlling factor in the drug release process. That is, the release rate of CFT through the vehicle network to the external medium is significantly dependent on the rate of molecular diffusion of the CS/TPP/HA nanoparticulate system. The CS/TPP/HA NP prevented the release of most of the drug and granted a sustained release of CFT.

### 2.3. In Vitro CFT Permeation Studies

**Figure 2:**
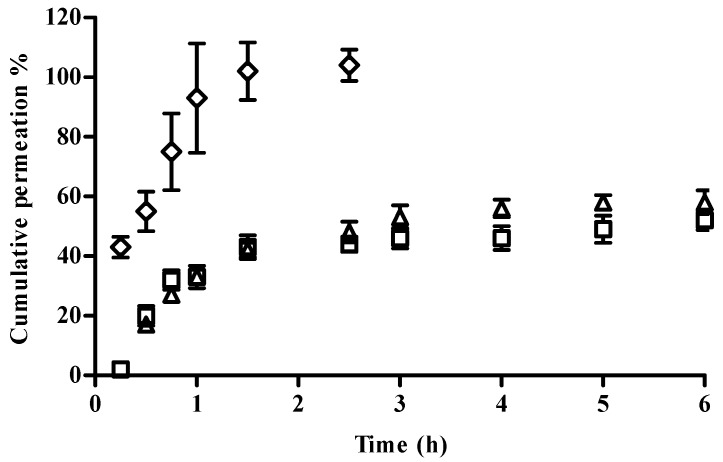
Cumulative permeation profile of NP in solution (□), NP in hydrogel (∆), and a free CFT solution (◊) in 10 mM PBS pH 7.4 through a cellulose membrane at 37 °C (mean ± SD, *n* = 6).

The determination of the permeation profile of CFT from the formulations was carried out in Franz diffusion cells using cellulose membranes. The cumulative amounts of CFT permeated through the membrane are given in Figure 2. No differences in CFT permeation profiles were obtained with NP in solution or in hydrogel, which presented similar permeation fluxes and *K*p (Table 4). The results clearly showed the difference between the permeation of free CFT when compared to the nanoencapsulated drug. The former presented a faster permeation flux, with around 50% of CFT permeated in the first hour, and complete permeation was reached within less than 2 h. The observation of these permeation profiles from NP suggests an extended drug release, which could be advantageous in long-term treatments.

### 2.4. Mucoadhesion Studies

**Figure 3:**
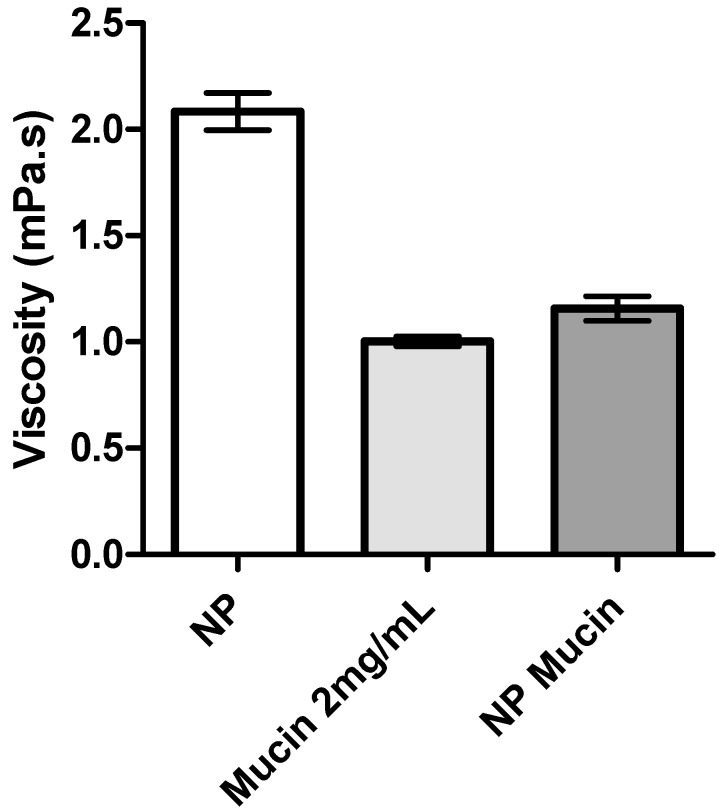
Viscosity values of mucin dispersion (0.2%, *w*/*v*) alone and of CS/TPP/HA NP isotonic solution before (NP) and after incubation with mucin (NP Mucin) (mean ± SD, *n* = 3).

**Figure 4:**
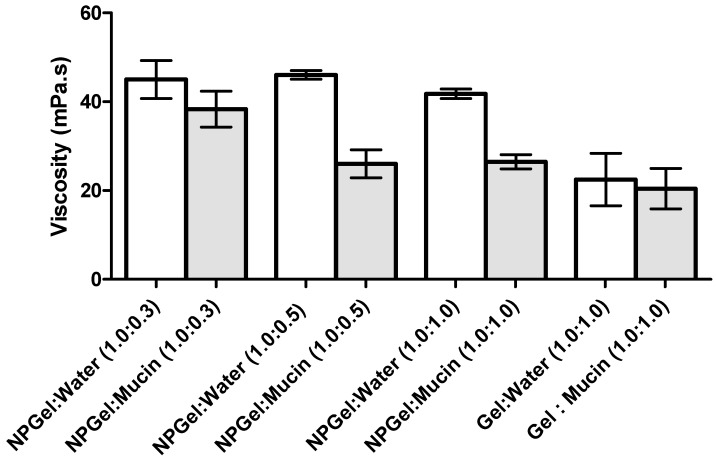
Viscosity values of HPMC 0.75% (*w/v*) hydrogel alone and containing nanoparticles (NPgel) before and after incubation with mucin at different polymer:mucin ratios (mean ± SD, *n* = 3).

Biological fluids contain important amounts of proteins and enzymes, which can influence the stability of NP formulations. The interaction and stability of NP-eye drop formulations in the presence of mucin was determined by measuring the viscosity of mucin dispersion before and after incubating with the eye drop formulation. The addition of mucin dispersion to the formulations led to a significant decrease in the viscosity of the medium (Figure 3 and Figure 4). 

The formation of interactions between polymer and mucin was also determined by analyzing the changes in the viscosity of the mixtures as a function of polymer:mucin weight ratio (Figure 4). As mucin concentration increases, there is a decrease in viscosity. The mucin solution used in this study was prepared with purified water; in this medium, the partially ionized mucin chains repel each other, causing a more extended polymer conformation which is available to interact with polymers to a higher degree [21].

**Figure 5:**
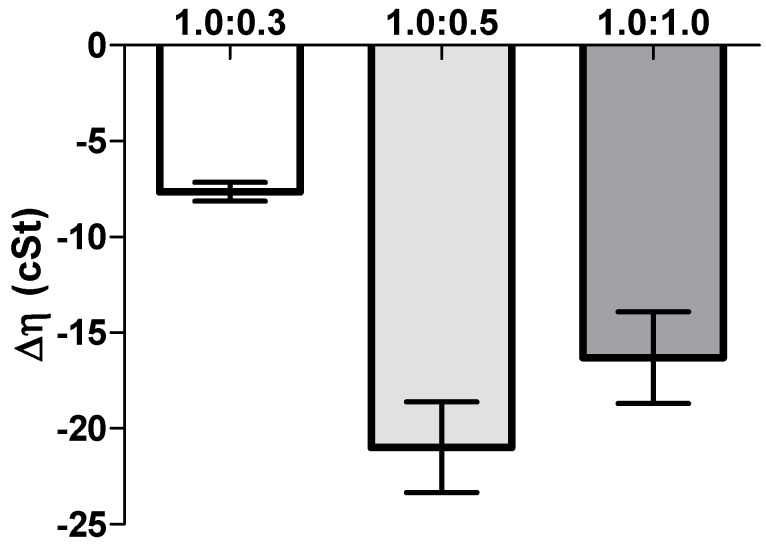
Rheological synergism (cSt) for NP eye drop formulation (HPMC 0.75%, *w*/*v*) mixed with increasing mucin amounts (mean ± SD, *n* = 3).

The rheological synergism has been recommended as an in vitro parameter to determine the mucoadhesive properties of polymers [22]. Although there are positive values in the viscosity of the solutions NP-gel-mucin at different polymer:mucin ratios, such viscosities are lower than the value (ηNPpol + ηmuc). The present results demonstrated that a negative rheological synergism occurs for all the tested ratios, but the most negative was observed for the ratio 1:0.5 (Figure 5).

Changes in ZP upon NP incubation with mucin dispersion also suggested that interactions between mucin and NP were present, causing a marked reduction in ZP after adding mucin (Table 5). The results also suggested a limited interaction between the HPMC hydrogel and mucin with a slight decrease in the gel viscosity, and ZP practically unchanged (Table 5).

### 2.5. Antimicrobial Activity

The susceptibility of *P. aeruginosa* to CFT—either free or encapsulated in NP—was determined using the agar diffusion method (Table 6). Apparently, the antimicrobial activity of CFT decreased upon encapsulation in NP, and when the NP were incorporated into the hydrogel formulation. However, encapsulation clearly has a stabilizing effect on CFT, showing no antimicrobial activity loss after 2 months. On the other hand, the inhibition zone obtained with NP hydrogel formulation was smaller than that of the NP isotonic solution, and HPMC gel acts as an additional barrier for drug diffusion. As CS has antimicrobial properties [13,23], plain NP were also tested, showing that the CS concentration used in NP was not enough to inhibit the bacterial growth of *P. aeruginosa*, as no inhibition zone was detected (Table 6). 

The minimum inhibitory concentration (MIC) determination of free CFT and CFT-loaded NP, performed using the microtiter plate assay support the data from the agar diffusion test. The MIC value of the CFT-loaded NP in isotonic solution and free CFT were found to be 1.56 µg/mL for *P. aeruginosa*, indicating that antibacterial activity was preserved when the drug was loaded into CS/TPP/HA NP. The MIC value of the NP gel formulation was significantly higher (3.13 µg/mL), probably for the same reason described above. Nevertheless, the prepared nano-formulations preserved the antibacterial efficiency of CFT and can be potentially used to treat ocular diseases.

### 2.6. In Vitro Cell Assays

#### 2.6.1. Cell Viability

**Figure 6:**
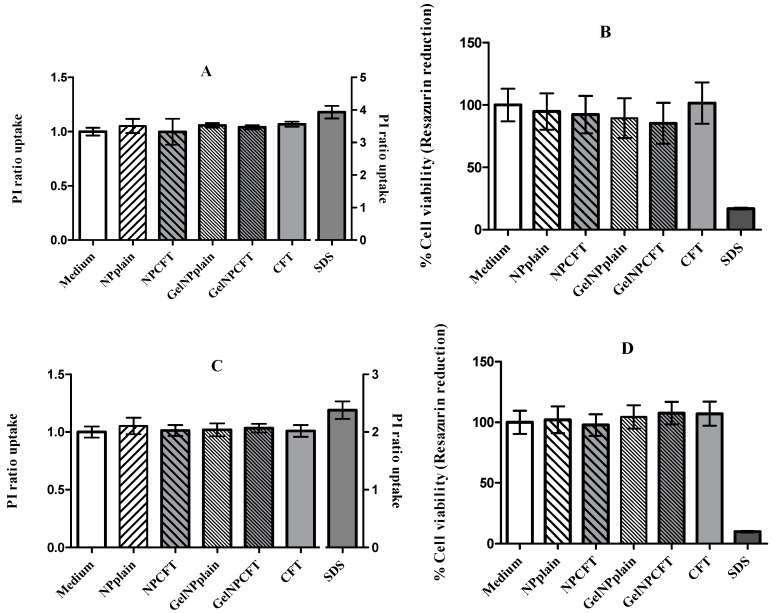
Cell viability of HEK293T (**A**,**B**) and ARPE-19 (**C**,**D**); cell lines were exposed for 24 h to 200 µg/mL of formulations. (**A**,**C**) propidium iodide (PI) ratio uptake; (**B**,**D**) rezasurin reduction (mean ± SD, *n* = 6).

The cell viability of the ARPE-19 and HEK 293T cell lines, as measured by a metabolic and membrane integrity assay, suggested that the tested formulations were not toxic at concentrations below 200 µg/mL (Figure 6). These findings are in agreement with previously published data where the tested formulation of NP showed 100% of cell viability [15].

#### 2.6.2. Oxidative Stress Assay

**Figure 7:**
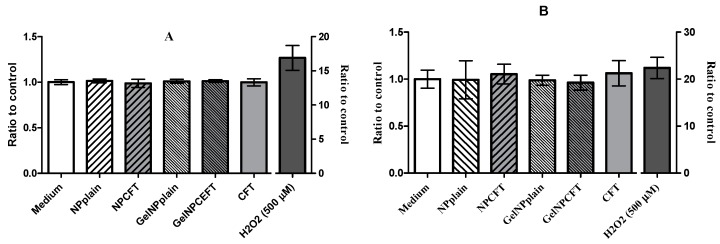
Production of reactive oxygen species (ROS). Cells exposed 24 h to 200 µg/mL of formulations: (**A**) HEK293T cell line; (**B**) ARPE-19 cell line (mean ± SD, *n* = 6).

The ability of NPs to induce intracellular oxidant production in HEK 293T and ARPE-19 cells was assessed using 2′,7′-dichlorofluorescein (DCF) fluorescence. All tested samples did not significantly increase (*p* < 0.05) the intracellular reactive oxygen species (ROS) production in both cell lines after 24 h of exposure (Figure 7).

## 3. Discussion

The design and characterization of a novel CS/TPP/HA nanoparticulate system containing CFT aiming to interact with the ophthalmic mucosal barrier and favoring the transport of the antibiotic is described in the present work. Polymeric NP are a promising strategy, as they can encapsulate drug molecules, confer protection against premature degradation, and are also able to increase ocular penetration and bioavailability due to their small particle size and—in some cases—mucoadhesive properties [16]. 

The CS/TPP/HA NPs were prepared by ionotropic gelation—a rapid and mild method based on the spontaneous formation of complexes between CS and TPP/HA. The NP formation occurs due to the establishment of hydrogen and ionic bonds between the positively-charged amino groups of CS and negatively-charged phosphate groups of TPP and carboxyl groups of HA. The TPP is also responsible for the formation of very organized structures [15,24]. This method usually forms small (200–500 nm), spherical NP, resulting in more efficient drug transport trough the biological barriers [23,25]. To improve encapsulation efficiency, the pH of the TPP solution was adjusted to 7.5 to guarantee that CFT molecules had a negative charge when added to the acidic CS solution. In these conditions, CFT molecules can bind to the positively-charged groups of CS. 

The herein described NPs presented a particle size of around 350 nm with high positive values of ZP, which indicates good stabilization of the colloidal systems due to greater electrostatic repulsion between the particles. In addition, also reveals the NP surface is mostly composed of CS. The positive charge of the particles is desirable to prevent particle aggregation and promote stronger electrostatic interaction with the negatively charged sialic acid residues of mucin in the eye surface [25,26]. Similar results were achieved in other studies, with CS/HA NP encapsulating other drug molecules. For example, Nasti et al. [24] obtained CS/TPP NP coated with HA with mean particle size of 300 nm, whereas De la Fuente [16] used CS/HA NP of 300–400 nm to encapsulate bovine serum albumin (BSA) and cyclosporine A as model protein drugs. Concerning ZP, the results described herein are in line with those reported in the literature for similar NP formulations [15,16,23]. 

The in vitro release studies performed with CS/TPP/HA NP showed a prolonged release of CFT. First there was an initial fast release of CFT, possibly due to desorption of drug molecules located at the surface of NP, followed by the diffusion of free drug. After the initial fast release, a slower sustained release occurred, which can be attributed to the slow diffusion of the encapsulated drug out of the polymeric matrix of NP into the release medium. The drug release mechanism from NP formulations is described in the literature as being trough diffusion, desorption, and matrix degradation. The drug release profile obtained is similar to those found in the literature for CS/HA NP. Yang et al. [27] also observed an initial fast drug release followed by sustained release of curcumin during 36 h. The HPMC gel can entrap CFT molecules, resulting in a prolonged drug release, suggesting that the diffusion rate of CFT from NP can be modified by changing the viscosity of the vehicle. However, it would be expected that drug permeation from the gel formulation would be lesser than the permeation from the NP formulation because the higher viscosity of the gel results in a more compact polymer matrix. This would theoretically reduce the diffusion of CFT from the NP [28].

Another important aspect in ophthalmic formulations is the interactions between the nanoparticles and mucins present in the ocular surface. Surprisingly, the mucoadhesion studies revealed a negative interaction; i.e., a negative rheological synergism was obtained at different NP gel:mucin weight ratios, with the lowest value observed for the 1:0.5 mixture. The results may point out the formation of products resulting from the interaction (NP gel)–mucin with a stoichiometry of the interaction product close to that weight ratio. 

Other authors found a synergic increase in viscosity when mixing hydrated CS with mucin. However, in those works, the mucin concentration (% *w*/*v*) was higher than the one used herein [21,22]. A synergistic increase in viscosity has been observed when an excess of mucin is present [29], while other authors [5] showed no viscosimetric changes in the mucin-CS-NP dispersion. However, in the present study, NP were prepared with low-molecular weight (LMW) CS, which probably has enough chain flexibility to enable the mucin interactions, facilitating the NP penetration into the branching sugars of mucin. Besides, the interactions between amino groups on CS and active groups on the sugar lateral-chains of mucin may occur and result in stronger mucoadhesivity [30].

Additionally, Rossi et al. [21,29] suggest that a negative interaction parameter can occur due to the adsorption of mucin onto the NP, and eventually a slight aggregation problem. This is in line with the ZP results, which showed a decrease of the NP values after the addition of mucin; the decrease in ZP values can be attributed to the ionic interactions and subsequent adsorption of negatively charged mucin particles onto the surface of positively charged chitosan NPs. HPMC gel did not show significant changes in viscosity or ZP values when in contact with the mucin solution, which may be due to the lack of interactions between the cellulose polymer and mucin. As HPMC is neutral, it does not have ionized groups to interact with the sialic acid residues of mucin. These observations suggest that HPMC’s ability to increase the drug retention time in the eye is more related to its capacity to increase viscosity rather than to any interactions with mucus [31]. In conclusion, the produced NP are able to interact with mucin, leading to a significant alteration of the viscosity of NP dispersions, increasing the residence time of the NP on the eye surface, which improves the drug absorption and can reduce the frequency of administration [22]. 

Importantly, the prepared CS/TPP/HA NP preserved the antibacterial activity of CFT and can potentially be used to treat ocular diseases. 

Finally, in vitro studies showed that NPs were not toxic to the tested cell lines, in agreement with previously published data where the NP formulation showed 100% cell viability [15]. 

## 4. Materials and Methods

### 4.1. Materials

Low-molecular weight (LMW) chitosan (CS) with degree of deacetylation (DD) 75–85%, sodium chloride, hydrochloric acid, hydroxypropylmethyl cellulose (HPMC), 10 mM phosphate-buffered saline (PBS), polyvinyl alcohol (PVA), and mucin from porcine stomach were obtained from Sigma-Aldrich (Irvine, UK). Sodium tripolyphosphate (TPP) was obtained from AppliChem (Darmstadt, Germany). Hyaluronic acid (HA) was a kind gift from Soliance (Paris, France), and ceftazidime (CFT) was offered by Combino Pharm Portugal. 

Purified water was of Milli-Rx quality (Merck Millipore, Darmstadt, Germany). All other reagents and solvents were of the purest grade available, and generally were used without further treatment. 

Tryptic soy broth and tryptic soy agar were obtained from Biokar (Pantin, France). 

*Pseudomonas aeruginosa* (ATCC 9027), HEK293T (ATCC^®^ CRL-11268™), and ARPE-19 (ATCC^®^ CRL-2302™) cell lines were obtained from the American Type Cell Culture collection (Manassas, VA, USA).

Cell culture media and supplements were from Gibco (ThermoFisher Scientific, Paisley, UK).

### 4.2. Methods

#### 4.2.1. Formulation of CS/TPP/HA NP Formulations

The CS/TPP/HA nanoparticles (NP) were prepared using an ionic gelation technique, as previously described elsewhere [32]. Firstly, CS LMW 1% (*w*/*v*) was dissolved in a 0.5% (*v*/*v*) acetic acid aqueous solution. Then, the aqueous solution was further diluted to prepare a CS solution at 2.5 mg/mL and the pH value was corrected to 5 using 1 N sodium hydroxide. After that, solutions of HA (HA 50 at 10 mg/mL), NaCl 0.9%, and CFT (40 mg/mL) were incorporated in the TPP solution (1.5 mg/mL at pH 7.5). The NPs were obtained upon the dropwise addition of this solution (TPP-HA-CFT-NaCl) to the CS solution under constant stirring (300 rpm). The final isotonic solution remained under magnetic stirring at room temperature for 15 min to ensure system stabilization. All of the previously mentioned solutions were prepared using purified water. 

The eye preparations were prepared by dissolving 0.75% (*w*/*v*) of (hydroxypropyl) methyl cellulose (HPMC) in the CS/TPP/HA NP nanoparticle suspension. The formulation was maintained under magnetic stirring for 24 h. The HPMC was previously characterized in terms of: viscosity using a rotational viscometer (Brookfield, Vernon Hills, IL, USA) with the spindle number 21; pH value (pH WTW meter, WTW GmbH, Dinslaken, Germany); osmolality (Osmometer K-7400, Knawer, Berlim, Germany); and zeta potential (ZP), using electrophoretic mobility (Nanosizer Nano Z, Malvern Instruments, Malvern, UK). 

#### 4.2.2. Nanoparticle Characterization

##### Particle Size, Size Distribution, and Zeta Potential

The particle size and size distribution (polydispersity index, PDI) were determined by dynamic light scattering using a Zetasizer Nanoseries Nano S (Malvern Instruments, Malvern, UK). The samples were suitably diluted in filtered 0.22 µm purified water and analyzed at 25 °C. The ZP was measured using a Zetasizer Nanoseries Nano Z (Malvern Instruments, Malvern, UK). All measurements were performed in triplicate, and the results are shown as the mean ± standard deviation (SD).

##### Quantification of CFT, Encapsulation Efficiency, and Drug Loading

The CFT encapsulation in NP was determined after centrifugation of 1 mL of the NP samples using an Eppendorf centrifuge (12,000× *g*, 15 min). The amount of free drug in the aqueous phase was measured in the supernatant using UV-Visible spectrophotometry, at 256 nm in a microplate spectrophotometer reader (FLUOstar Omega. BMGLabtech, Ortenberg, Germany). A calibration curve was constructed using different CFT concentrations between 250 µg/mL and 1.95 µg/mL. The supernatant of unloaded nanoparticles was used as basic correction. The CFT encapsulation efficiency (EE) and drug loading (DL) of CFT in NP were obtained using the following equations:

(1) $$\%\;EE=\frac{\left(W_{t}-W_{f}\right)}{W_{t}}\times 100$$

(2) $$\%\;DL=\frac{\left(W_{t}-W_{f}\right)}{W_{\mathrm{np}}}\times 100$$

where *W*_f_ is the amount of free CFT and *W*_t_ is the total amount added to the NP formulation and *W*_np_ is the weight of the nanoparticles.

##### In Vitro Ceftazidime Release Studies

The release of CFT from the NP was studied by a dialysis method. One milliliter of NP and eye drop formulations were introduced inside a tube of semi-permeable dialysis membrane (300 kDa) and then dipped in the receptor compartment containing 15 mL of phosphate buffer release medium (10 mM in pH 7.4). The receptor compartments were closed to prevent dissolution of the release medium and incubated with continuous horizontal shaking at 37 °C at 300 rpm. At regular time intervals, samples were withdrawn and the same volume was immediately replaced with fresh PBS solution maintained at the same temperature. All studies were conducted in triplicate. The amount of CFT released was evaluated by UV-spectrophotometry in a microplate reader at 256 nm. 

In order to investigate the mechanism of CFT release from the NP, the in vitro release data obtained were analyzed by fitting to five different kinetic models: zero-order (Equation (3)), first-order (Equation (4)), Hixson–Crowell (Equation (5)), Higuchi (Equation (6)) and Korsmeyer–Peppas (Equation (7)):

(3) $$Q_{t}=Q_{0}+K_{0}t$$

(4) $$\log Q_{t}=\log Q_{0}-\frac{K_{1}t}{2.303}$$

(5) $$\sqrt[{3}]{Q_{0}}-\sqrt[{3}]{Q_{t}}=K_{\mathrm{HC}}$$

(6) $$Q_{t}=K_{H}\sqrt{t}$$

(7) $$\frac{Mt}{M\infty }=K_{\mathrm{KP}}t^{n}$$

where *Q*_0_ is the initial amount of CFT. *Q*_t_ is the cumulative amount of drug release at time t. *K*_0_ is the zero-order release constant, *K*_1_ is the first-order release constant, *K*_HC_ is the Hixson–Crowell constant, *K*_H_ is the Higuchi constant, *K*_KP_ is the Korsmeyer–Peppas constant, and *n* is an exponent characterizing the release mechanism. The model that best fits the experimental data was selected based on the highest correlation coefficient (*r*^2^) values.

##### In Vitro Release Studies

Drug release was investigated with Franz-type diffusion cells with cellulose membranes separating the donor and receptor phase. The receptor phase was composed of approximately 4 mL phosphate buffer at pH 7.4. The donor phase of the Franz cells was filled with 200 μL of each sample: NP solution, NP eye drop formulation, and free CFT. The Franz cells were incubated at 37 °C with magnetic stirring at 300 rpm. Over a period of 6 h at predefined time intervals, a sample (200 μL) of the receptor solution was collected for the determination of drug released and replaced with equal volume of fresh phosphate buffer maintained at the same temperature. All experiments were carried out using five cells per formulation and performed under sink conditions. The samples were analyzed by UV-spectrophotometry in a microplate reader at 256 nm.

The percentage of cumulative amount of CFT permeated through the membrane (% µg/cm^2^) was plotted as a function of time and determined using Equation (8):

(8) $$\%\;Q_{t}=\frac{V_{r}\times C_{t}+\sum_{t=0}^{t-1}V_{s}\times C_{i}}{S}\times \frac{100}{\mu \mathrm{gCFT}}$$

where *C*_t_ is the CFT concentration of the receptor solution at each sampling time, *C*_i_ is the concentration at each sampling time, *V*_r_ is the volume of the receptor solution and *V*_s_ the volume of the sample. The *S* value is the membrane area (1 cm^2^). The weight of CFT (µg) corresponds to the 200 µL of sample CFT solution. The fluxes and the experimental *K*_p_ were achieved by fitting the curves of the graph of the cumulative amount of drug permeated through the membrane per unit surface area vs. time (*Q*_t_ (µg/cm^2^) vs. time (h)) in the linear region. The fluxes (*J*, µg/cm^2^/h) were calculated from the slope of the linear portion of the plot and the permeability coefficients (*K*_p_, cm/h) were obtained by dividing the flux (*J*) by the initial drug concentration (*C*_0_) present in the donor phase. It was assumed that in sink conditions the drug concentration in the receptor phase is insignificant compared to the one that is present in the donor phase.

##### Mucoadhesion Studies

The interaction between mucin and NPs formulations was assessed using two in vitro methods: viscosity and zeta potential measurements.

##### Viscosity Measurements

To study the effect of mucin interaction, the viscosity of three different NP-gel:mucin weight ratios were considered: 1:0.3, 1:0.5, and 1:1. For comparison, the viscosity of NP isotonic solutions (without mucin diluted in purified water at the same ratio as previously described) was also calculated. Additionally, the viscosity of the mucin dispersion in water (2 mg/mL) was assessed. The measurements were repeated at least three times. 

The viscosity of all the samples of eye drop formulations (NP-HPMC 0.75%) with or without mucin (2 mg/mL) addition and also the mucin dispersion in water were measured at 25 °C using a capillary Ostwald Viscometer. The viscosity component due to bioadhesion or rheological synergism parameter (∆η) was calculated as follows [25]:

∆η = η_mix_ − (η_muc_ + η_pol_) (9)

where η_mix_ is the viscosity of the NP-HPMC-mucin mixture (mPa s); η_pol_ is the viscosity of the NP-HPMC gel having the same concentration as in the mixture. η_muc_ is the viscosity of mucin dispersion having the same concentration as in the mixture (mPa s).

##### Zeta Potential Measurements

The second method evaluated the effect of mucin on the ZP of the formulations, which was measured before and after the incubation with mucin as described in the first subsection of Section 4.2.2.

##### Determination of Antimicrobial Activity

The antimicrobial activity was evaluated using two methods: the agar diffusion method and the microtitre plate antibacterial assay. In the agar diffusion method, tryptic soy agar (TSA) was used as growth medium with a concentration of 15 g/L agar-agar. This solution was sterilized in autoclave at 121 °C, 15 min. Under aseptic conditions, the TSA containing the microorganism *Pseudomonas aeruginosa* (ATCC 9027) was placed in a Petri dish and solidified. Sterile paper discs (6 mm; Thermo Scientific™ Oxoid™, Dardilly, UK) were loaded with 15 μL of NP formulations in triplicate positioned on the solid agar. To evaluate a possible loss in the antimicrobial activity with time, samples of NP with 1 day and with 2 months were also tested. The Petri dishes were incubated at 35 ± 2 °C overnight. In this assay, the parameter used to study the antimicrobial activity is the mean diameter of the inhibition zone formed around the disc after incubation. The diameters were measured using a Vernier caliper. 

The microtitre plate antibacterial assay was adapted from the method previously described by Sarker et al. [33] and according to the guidelines of Clinical and Laboratory Standards Institute (CLSI) by broth microdilution method [34]. The bacterial culture of *P. aeruginosa* was prepared in the tryptic-casein soy broth (TSB). After 24 h of incubation, the optical density of *P. aeruginosa* was recorded at 600 nm, and various dilutions were made with aseptic techniques until the optical density was between 0.5 and 1.0. The dilution factor was obtained (=300) after that the dilution was carried out to achieve a concentration of 5 × 10^5^ CFU/mL.

We studied the antimicrobial effect of NP formulations with 1 day and 2 months, the NP gel formulation, and free CFT. The final concentration of free CFT and CFT-loaded NP used in this assay was 100 µg/mL. A sterile 96-well plate was prepared under aseptic conditions. The first row was filled with 100 µL of TSB, the negative control. All other wells were filled with 50 µL of TSB, including the last row containing 50 µL *P. aeruginosa,* which is the positive control. The samples were subject to serial dilutions using a multichannel pipette. Finally, the bacterial suspension was added to each well to achieve a concentration of 5 × 10^5^ CFU/mL. The plate was incubated at 35 ± 2 °C overnight. The turbidity change was first assessed visually. A vital dye Alamar Blue was added to all wells in the plate and incubated for 3 h. The color changes from blue to pink were recorded as positive. To confirm the positive and negative results, the wells’ fluorescence intensity was measured in a microplate spectrophotometer reader (FLUOstar Omega. BMGLabtech, Ortenberg, Germany). The first concentration with no sign of bacterial growth was taken as the MIC value.

#### 4.2.3. In Vitro Cell Assays

##### Cell Viability

The cytotoxicity was assessed using general cell viability endpoint resazurin reduction (7-hydroxy-3H-phenoxazin-3-one 10-oxide) (Alamar Blue) assay and propidium iodide (PI) dye exclusion assays [35,36]. Resazurin is a weakly fluorescent blue dye that is reduced by viable cells into the pink-colored and highly red fluorescent resorufin. Propidium iodide (PI) is a red fluorescent probe that is a cell membrane impermeant, and therefore only penetrates membrane integrity-compromised cells. When PI does gain access to nucleic acids and intercalates them, its fluorescence increases dramatically and it is therefore used to identify membrane integrity-compromised cells. 

Cell viability was assessed after 24 h of incubation with different concentrations of the formulations. The day before experiment HEK293T (human embryonic kidney cell line, ATCC^®^ CRL-11268™) and ARPE-19 (human retinal pigment epithelial cell line, ATCC^®^ CRL-2302™) cell lines were seeded in sterile flat-bottom 96-well tissue culture plates (Greiner, Kremsmünster, Austria) in RPMI 1640 culture medium (ThermoFisher Scientific, Paisley, UK), supplemented with 10% fetal bovine serum (ThermoFisher Scientific, Paisley, UK), 100 units/mL of penicillin G (sodium salt) (Life Technologies, Paisley, UK), 100 μg/mL of streptomycin sulfate (ThermoFisher Scientific, Paisley, UK), and 2 mM l-glutamine (ThermoFisher Scientific, Paisley, UK) at a cell density of 2 × 10^5^ cells/mL. Cells were incubated at 37 °C and 5% CO_2_.

On the next day, medium was replaced by fresh medium containing the different samples to be analyzed. Each concentration was tested in six wells per plate. Cells were incubated for 48 h. The negative control was the culture medium, and positive control was sodium dodecyl sulfate (SDS) at 0.1 mg/mL. After the time of exposure, the medium was replaced by 0.3 µM propidium iodide in culture medium (stock solution 1.5 mM in DMSO, diluted with culture medium 1:5000). Fluorescence was measured (excitation: 485 nm; emission: 590 nm) in microplate reader (FLUOstar Omega, BMGLabtech, Ortenberg, Germany) and then the Alamar Blue assay was performed. Medium was replaced by medium containing 5 mM of resazurin. The cells were further incubated for 3 h and the fluorescence at 530 nm of excitation wavelength and 590 nm of emission wavelength was measured in a fluorescence microplate reader (FLUOstar Omega, BMGLabtech, Ortenberg, Germany).

The relative cell viability (%) compared to control cells was calculated by
[Fluorescence]sample/[Fluorescence]control × 100 for the Alamar Blue assay and
[Fluorescence]sample/[Fluorescence]control for PI uptake assay.

##### Oxidative Stress Assay

The intracellular reactive oxygen species (ROS) production was determined using the 2′,7′-dichlorodihydrofluorescein diacetate (H2DCFDA) dye. H2DCFDA is a stable non-fluorescent molecule that is hydrolyzed by intracellular esterases to non-fluorescent 2′,7′-dichlorofluorescein (DCF) which is rapidly oxidized in the presence of oxygen radicals to a highly fluorescent compound (DCF) [36,37]. Cultures at the same cell density for cell viability assay of ARPE-19 and HEK 293T cells grown in 96-well plates were incubated, after exposed for 24 h at different samples 30 min with 20 μM of H2DCFDA (ThermoFisher Scientific, Paisley, UK) in the dark at 37 °C. The medium was then removed and fresh medium was added. Hydrogen peroxide solution (AppliChem, Darmstadt, Germany) was used as a positive control for the induction of ROS in cells and media alone as a negative control. ROS levels were determined at excitation 485 nm and emission 520 nm wavelengths using a florescence microplate reader (FLUOstar BMGLabtech, Ortenberg, Germany). Data from six replicates are reported as relative fluorescence units (RFU) percentage and expressed as mean fluorescence ratio (fluorescence of exposed cells/fluorescence of unexposed control from the same experiment).

#### 4.2.4. Statistical Data Analysis

The data was expressed as mean and standard deviation (mean ± SD) of experiments (*n* = 6). Statistical evaluation of data was performed using one-way analysis of variance (ANOVA). Tukey–Kramer multiple comparison test (GraphPad PRISM 5 software, La Jolla, CA, USA) was used to compare the significance of the difference between the groups, and a *p* < 0.05 was accepted as significant.

## 5. Conclusions

To our knowledge, this is the first time that mucoadhesive CS/TPP/HA NP were prepared to encapsulate CFT for the treatment of serious eye infections caused by *Pseudomonas aeruginosa,* such as bacterial keratitis. The developed NP presented physicochemical and pharmaceutical characteristics suitable for topical ocular administration, while preserving the antimicrobial activity of CFT. In addition, the nanoformulation presented relevant mucoadhesive properties, interacting with mucin, which is desirable to improve the absorption and effectiveness of the antibiotics. This NP arises as a promising drug delivery system for topical opthalmic antibiotic therapy, increasing drug residence in the eye.

## Figures and Tables

**Table 1:** Critical quality attributes to the (hydroxypropyl) methyl cellulose (HPMC) 0.75% (*w*/*v*) hydrogel.

Parameter	HPMC 0.75% (*w*/*v*)
pH	7.22
Osmolality	292 mOsm/Kg
Zeta Potential	−3.22 ± 0.88 mV
Viscosity	54.5 mPa s

**Table 2:** Particle size, polydispersity index (PDI), zeta potential (ZP), encapsulation efficiency (EE) and drug loading (DL) of chitosan (CS)/sodium tripolyphosphate (TPP)/hyaluronic acid (HA) nanoparticles (mean ± SD, *n* = 3). CFT: ceftazidime; NP: nanoparticles.

Formulation	Size (nm)	PDI	ZP (mV)	EE (%)	DL (%)
Empty NP	395 ± 4	0.25 ± 0.02	+46 ± 1	n.a.	n.a.
NP loaded with CFT	362 ± 35	0.14 ± 0.00	+44 ± 1	41 ± 4	1.7 ± 0.1

n.a.: not applicable.

**Table 3:** Mathematical models and respective parameters (correlation coefficients and release constants) obtained from the fitting of the experimental data corresponding to a CFT release from CS/HA/TPP nanoparticles. The release constants were calculated from the slopes of the respective plots.

Formulation	Zero-Order		First-Order		Higuchi		Hixson–Crowell		Korsmeyer–Peppas		
	*r*^2^	*k*_0_ (µg/h)	*r*^2^	*k*_1_ (h^−1^)	*r*^2^	*k*_H_ (h^−0.5^)	*r*^2^	*k*_HC_	*r*^2^	*k*_KP_ (h*^n^*)	*n*
NP	0.739	8.96	0.829	0.14	0.937	21.74	0.906	2.99	0.949	0.70	0.266
NP Gel	0.659	5.92	0.689	0.07	0.875	14.69	0.698	2.04	0.889	0.71	0.418

*r*^2^: correlation coefficient; *k*_0_: zero-order release constant; k_1_: first-order release constant; *k*_H_: Higuchi constant; *k*_HC_: Hixson–Crowell constant; *k*_KP_: Korsmeyer–Peppas constant; *n*: release mechanism exponent.

**Table 4:** Permeation flux and *K*p of CFT through a cellulose membrane for NP in solution, NP in hydrogel, and free CFT solution. (mean ± SD, *n* = 6).

Formulation	*J*ss (µg/(cm^2^h))	Experimental *K*p (cm/h)
NP isotonic solution	27.8 ± 3.3	0.034 ± 0.004
NP gel	26.6 ± 1.0	0.033 ± 0.001
Free CFT	73.5 ± 18.0	0.092 ± 0.020

*J*ss—flux at steady-state; *K*p—permeability coefficient.

**Table 5:** Zeta potential of, NP, NP gel, and HPMC hydrogel 0.75% (*w/v*) upon dilution 1:1 with water or mucin dispersion 2 mg/mL (mean ± SD, *n* = 3).

Formulation	Zeta Potential (mV)	
Solutions	Water	Mucin 2 mg/mL
Mucin 2 mg/mL	-	−31 ± 1
Empty NP	+60 ± 4	+12 ± 0
Empty NP eye drop formulation (HPMC 0.75%)	+36 ± 1	+7 ± 0
HPMC 0.75%	−14 ± 3	−9 ± 1

**Table 6:** Antimicrobial activity of free CFT, CFT-NP isotonic solution, and CFT-NP in hydrogel (*P. aeruginosa*)*.*

Formulation	Inhibition Zone Diameter (mm)	MIC (µg/mL)
NP isotonic solution	13.2	1.56
NP isotonic solution (2 months)	14.1	1.56
NP Gel	10.6	3.13
Free CFT	20.5	1.56
Plain NP	<6	-

## References

[1] Diebold Y., Calonge M. (2010). Applications of nanoparticles in ophthalmology. Prog. Retin. Eye Res..

[2] Paolicelli P., de la Fuente M., Sánchez A., Seijo B., Alonso M.J. (2009). Chitosan nanoparticles for drug delivery to the eye. Expert Opin. Drug Deliv..

[3] Wijesooriya C., Budai M., Budai L., Szilasi M., Petrikovics I. (2013). Optimization of liposomal encapsulation for ceftazidime for developing a potential eye drop formulation. J. Basic Clin. Pharm..

[4] Baba K., Tanaka Y., Kubota A., Kasai H., Yokokura S., Nakanishi H., Nishida K. (2011). A method for enhancing the ocular penetration of eye drops using nanoparticles of hydrolyzable dye. J. Control. Release.

[5] De Campos A.M., Diebold Y., Carvalho E.L.S., Sánchez A., Alonso M.J. (2004). Chitosan Nanoparticles as New Ocular Drug Delivery Systems: In Vitro Stability, In Vivo Fate, and Cellular Toxicity. Pharm. Res..

[6] Gaudana R., Jwala J., Boddu S.H.S., Mitra A.K. (2009). Recent Perspectives in Ocular Drug Delivery. Pharm. Res..

[7] Mitra A.K., Anand B.S., Sridhar D. (2005). Drug Delivery to the eye. Advances in Organ Biology.

[8] Achouri D., Alhanout K., Piccerelle P., Andrieu V. (2013). Recent advances in ocular drug delivery. Drug Dev. Ind. Pharm..

[9] Le Bourlais C., Acar L., Zia H., Sado P.A., Needham T., Leverge R. (1998). Ophthalmic drug delivery systems—Recent advances. Prog. Retin. Eye Res..

[10] Budai L., Hajdu M., Budai M., Grof P., Beni S., Noszal B., Klebovich I., Antal I. (2007). Gels and liposomes in optimized ocular drug delivery: Studies on ciprofloxacin formulations. Int. J. Pharm..

[11] Urtti A. (2006). Challenges and obstacles of ocular pharmacokinetics and drug delivery. Adv. Drug Deliv. Rev..

[12] Lehr C.-M., Bouwstra J.A., Schacht E.H., Junginger H.E. (1992). In vitro evaluation of mucoadhesive properties of chitosan and some other natural polymers. Int. J. Pharm..

[13] Ludwig A. (2005). The use of mucoadhesive polymers in ocular drug delivery. Adv. Drug Deliv. Rev..

[14] Almeida H., Amaral M.H., Lobão P., Silva A.C., Lobo J.M.S. (2014). Applications of polymeric and lipid nanoparticles in ophthalmic pharmaceutical formulations: Present and future considerations. J. Pharm. Pharm. Sci..

[15] De la Fuente M., Raviña M., Paolicelli P., Sanchez A., Seijo B., Alonso M.J. (2010). Chitosan-based nanostructures: A delivery platform for ocular therapeutics. Adv. Drug Deliv. Rev..

[16] De la Fuente M., Seijo B., Alonso M.J. (2008). Novel hyaluronan-based nanocarriers for transmucosal delivery of macromolecules. Macromol. Biosci..

[17] Contreras-Ruiz L., de la Fuente M., García-Vázquez C., Sáez V., Seijo B., Alonso M.J., Calonge M., Diebold Y. (2010). Ocular tolerance to a topical formulation of hyaluronic acid and chitosan-based nanoparticles. Cornea.

[18] Remington J., Gennaro A., Troy D.B. (2006). The Science and Practice of Pharmacy.

[19] Zambito Y., Colo G.D., Pignatello R. (2011). Polysaccharides as Excipients for Ocular Topical Formulations. Biomaterials Applications for Nanomedicine.

[20] Almeida H., Amaral M.H., Lobão P., Lobo J.M.S. (2014). In situ gelling systems: A strategy to improve the bioavailability of ophthalmic pharmaceutical formulations. Drug Discov. Today.

[21] Rossi S., Ferrari F., Bonferoni M.C., Caramella C. (2000). Characterization of chitosan hydrochloride-mucin interaction by means of viscosimetric and turbidimetric measurements. Eur. J. Pharm. Sci..

[22] Hassan E.E., Gallo J.M. (1990). A simple rheological method for the in vitro assessment of mucin-polymer bioadhesive bond strength. Pharm. Res..

[23] Silva N.C., Silva S., Sarmento B., Pintado M. (2013). Chitosan nanoparticles for daptomycin delivery in ocular treatment of bacterial endophthalmitis. Drug Deliv..

[24] Nasti A., Zaki N.M., de Leonardis P., Ungphaiboon S., Sansongsak P., Rimoli M.G., Tirelli N. (2009). Chitosan/TPP and Chitosan/TPP-hyaluronic Acid Nanoparticles: Systematic Optimisation of the Preparative Process and Preliminary Biological Evaluation. Pharm. Res..

[25] Mahmoud A.A., El-Feky G.S., Kamel R., Awad G.E.A. (2011). Chitosan/sulfobutylether-β-cyclodextrin nanoparticles as a potential approach for ocular drug delivery. Int. J. Pharm..

[26] Gonçalves C., Pereira P., Gama M. (2010). Self-assembled hydrogel nanoparticles for drug delivery applications. Materials.

[27] Yang L., Gao S., Asghar S., Liu G., Song J., Wang X., Ping Q., Zhang C., Xiao Y. (2015). Hyaluronic acid/chitosan nanoparticles for delivery of curcuminoid and its in vitro evaluation in glioma cells. Int. J. Biol. Macromol..

[28] Abrego G., Alvarado H., Souto E.B., Guevara B., Bellowa L.H., Parra A., Calpena A., Garcia M.L. (2015). Biopharmaceutical profile of pranoprofen-loaded PLGA nanoparticles containing hydrogels for ocular administration. Eur. J. Pharm. Biopharm..

[29] Rossi S., Ferrari F., Bonferoni M.C., Caramella C. (2001). Characterization of chitosan hydrochloride—Mucin rheological interaction: Influence of polymer concentration and polymer: Mucin weight ratio. Eur. J. Pharm. Sci..

[30] Thongborisute J., Takeuchi H. (2008). Evaluation of mucoadhesiveness of polymers by BIACORE method and mucin-particle method. Int. J. Pharm..

[31] Bravo-Osuna I., Noiray M., Briand E., Woodward A.M., Argüeso P., Martínez I.T.M., Herrero-Vanrell R., Ponchel G. (2012). Interfacial interaction between transmembrane ocular mucins and adhesive polymers and dendrimers analyzed by surface plasmon resonance. Pharm. Res..

[32] Cadete A., Figueiredo L., Lopes R., Calado C.C.R., Almeida A.J., Gonçalves L.M.D. (2012). Development and characterization of a new plasmid delivery system based on chitosan-sodium deoxycholate nanoparticles. Eur. J. Pharm. Sci..

[33] Sarker S.D., Nahar L., Kumarasamy Y. (2007). Microtitre plate-based antibacterial assay incorporating resazurin as an indicator of cell growth and its application in the in vitro antibacterial screening of phytochemicals. Methods.

[34] Cockerill F.R., Wikler M.A., Alder J., Dudley M.N., Eliopoulos G.M., Ferraro M.J., Hardy D.J., Hecht D., Hindler J., Patel J. (2012). Methods for Dilution Antimicrobial Susceptibility Tests for Bacteria That Grow Aerobically; Approved Standard.

[35] Gaspar D.P., Faria V., Gonçalves L.M.D., Taboada P., Remuñán-López C., Almeida A.J. (2016). Rifabutin-loaded solid lipid nanoparticles for inhaled antitubercular therapy: Physicochemical and in vitro studies. Int. J. Pharm..

[36] Graça D., Louro H., Santos J., Dias K., Almeida A.J., Gonçalves L., Silva M.J., Bettencourt A. (2017). Toxicity screening of a novel poly(methylmethacrylate)-Eudragit nanocarrier on L929 fibroblasts. Toxicol. Lett..

[37] Marto J., Ascenso A., Gonçalves L.M., Gouveia L.F., Manteigas P., Pinto P., Oliveira E., Almeida A.J., Ribeiro H.M. (2016). Melatonin-based Pickering emulsion for skin’s photoprotection. Drug Deliv..

